# Prospection of Potential Actions during Visual Working Memory Starts Early, Is Flexible, and Predicts Behavior

**DOI:** 10.1523/JNEUROSCI.0709-23.2023

**Published:** 2023-12-06

**Authors:** Rose Nasrawi, Sage E. P. Boettcher, Freek van Ede

**Affiliations:** ^1^Institute for Brain and Behavior Amsterdam, Department of Experimental and Applied Psychology, Vrije Universiteit Amsterdam, Amsterdam 1081 BT, The Netherlands; ^2^Department of Experimental Psychology, University of Oxford, Oxford OX2 6GG, United Kingdom

**Keywords:** action planning, EEG, neural oscillations, visual working memory

## Abstract

For visual working memory to serve upcoming behavior, it is crucial that we prepare for the potential use of working-memory contents ahead of time. Recent studies have demonstrated how the prospection and planning for an upcoming manual action starts early after visual encoding, and occurs alongside visual retention. Here, we address whether such “output planning” in visual working memory flexibly adapts to different visual-motor mappings, and occurs even when an upcoming action will only potentially become relevant for behavior. Human participants (female and male) performed a visual-motor working memory task in which they remembered one or two colored oriented bars for later (potential) use. We linked, and counterbalanced, the tilt of the visual items to specific manual responses. This allowed us to track planning of upcoming behavior through contralateral attenuation of β band activity, a canonical motor-cortical EEG signature of manual-action planning. The results revealed how action encoding and subsequent planning alongside visual working memory (1) reflect anticipated task demands rather than specific visual-motor mappings, (2) occur even for actions that will only potentially become relevant for behavior, and (3) are associated with faster performance for the encoded item, at the expense of performance to other working-memory content. This reveals how the potential prospective use of visual working memory content is flexibly planned early on, with consequences for the speed of memory-guided behavior.

**SIGNIFICANCE STATEMENT** It is increasingly studied how visual working memory helps us to prepare for the future, in addition to how it helps us to hold onto the past. Recent studies have demonstrated that the planning of prospective actions occurs alongside encoding and retention in working memory. We show that such early “output planning” flexibly adapts to varying visual-motor mappings, occurs both for certain and potential actions, and predicts ensuing working-memory guided behavior. These results highlight the flexible and future-oriented nature of visual working memory, and provide insight into the neural basis of the anticipatory dynamics that translate visual representations into adaptive upcoming behavior.

## Introduction

Working memory allows us to retain past visual information to guide and prepare for potential future action. For example, when driving a car, you may sequentially check your surroundings through your mirrors, and check the navigation system, before shifting your gaze back to the road. You can use working memory representations of the surrounding traffic and your route to anticipate and prepare for what you might do next. From the perspective that working memory is ultimately aimed at prospecting and guiding potential future behavior (Fuster and Bressler, 2012; Chatham and Badre, 2015; Myers et al., 2017; de Vries et al., 2020; van Ede and Nobre, 2023), a central question is when and how this preparation for future actions takes place alongside the encoding and retention of visual information in working memory.

Information in working memory often becomes relevant for guiding behavior several seconds after sensory encoding. Accordingly, future action plans may form gradually during the memory delay (cf., Rainer et al., 1999), similar to the type of action planning observed preceding voluntary movement (Pfurtscheller and Berghold, 1989; Stancák and Pfurtscheller, 1996), or alongside perceptual decision making (Donner et al., 2009; Lange et al., 2013). By contrast, a recent study (Boettcher et al., 2021) revealed how the consideration of an anticipated future action commenced early after visual encoding. This pattern of action encoding, or “output planning at the input stage,” occurred even in the face of an intervening task and predicted performance several seconds later (Boettcher et al., 2021). We build on this and other recent research demonstrating action planning alongside visual working memory (Schneider et al., 2017; van Ede et al., 2019; Ester and Weese, 2023; Henderson et al., 2022; Nasrawi and van Ede, 2022; Rösner et al., 2022) and address three important open questions.

First, aforementioned studies linking visual representations to manual actions have done so by associating specific visual features to the required response hand. We and others have done so specifically by linking leftward and rightward visual tilt (from a vertical reference) respectively to responses with the left-hand and right-hand (van Ede et al., 2019; Boettcher et al., 2021). If action encoding truly reflects the consideration of prospective task demands, it should be invariant to specific visual-motor mappings. In the current study, we therefore counterbalanced the tilt-response mapping to isolate the flexible prospection of future actions alongside visual working memory, independent of visual tilt.

Second, in complex everyday environments (such as our car-driving example above), we often need to hold multiple visual representations in working memory, that serve multiple potential future courses of action (Cisek and Kalaska, 2010; van Ede et al., 2019; Ester and Weese, 2023; Nasrawi and van Ede, 2022; van Ede and Nobre, 2023). Yet, to date, the described pattern of action encoding in working memory has only been demonstrated when just one visual stimulus was relevant for a single upcoming action that was certain to become relevant (Boettcher et al., 2021; see also Schneider et al., 2017). In the current study, we address whether the early prospection of a future memory-guided action also occurs after the encoding of visual stimuli that will potentially become relevant for future behavior.

Finally, in Boettcher et al. (2021), stronger encoding of a certain future memory-guided action was associated with better task performance. By here considering a situation with multiple potentially relevant memory items, we can address whether stronger encoding of a potential future action is generally associated with faster performance. Alternatively, potential-action encoding might give rise to a performance trade-off whereby stronger encoding of one potential future action benefits the readiness to act on the associated memory content, at the expense of another.

We show early prospection of future actions alongside sensory encoding in visual working memory that: (1) flexibly adapts to specific visual-motor mappings, (2) occurs both for certain and potential actions, (3) and facilitates decision times for the corresponding visual representation, at the expense of another.

## Materials and Methods

### Participants

Twenty-five healthy human adults (age: mean = 22.85, SD = 4.03; gender: 17 female, 8 male; four left-handed) participated in the experiment. All participants had normal, or corrected-to-normal vision, and none were excluded from the analyses. The experiment was approved for by the Research Ethics Committee of the Vrije Universiteit Amsterdam. Participants provided written informed consent before participating in the study, and were rewarded €10 or 10 research credits per hour for their participation.

### Experimental design and procedure

**Figure 1.**
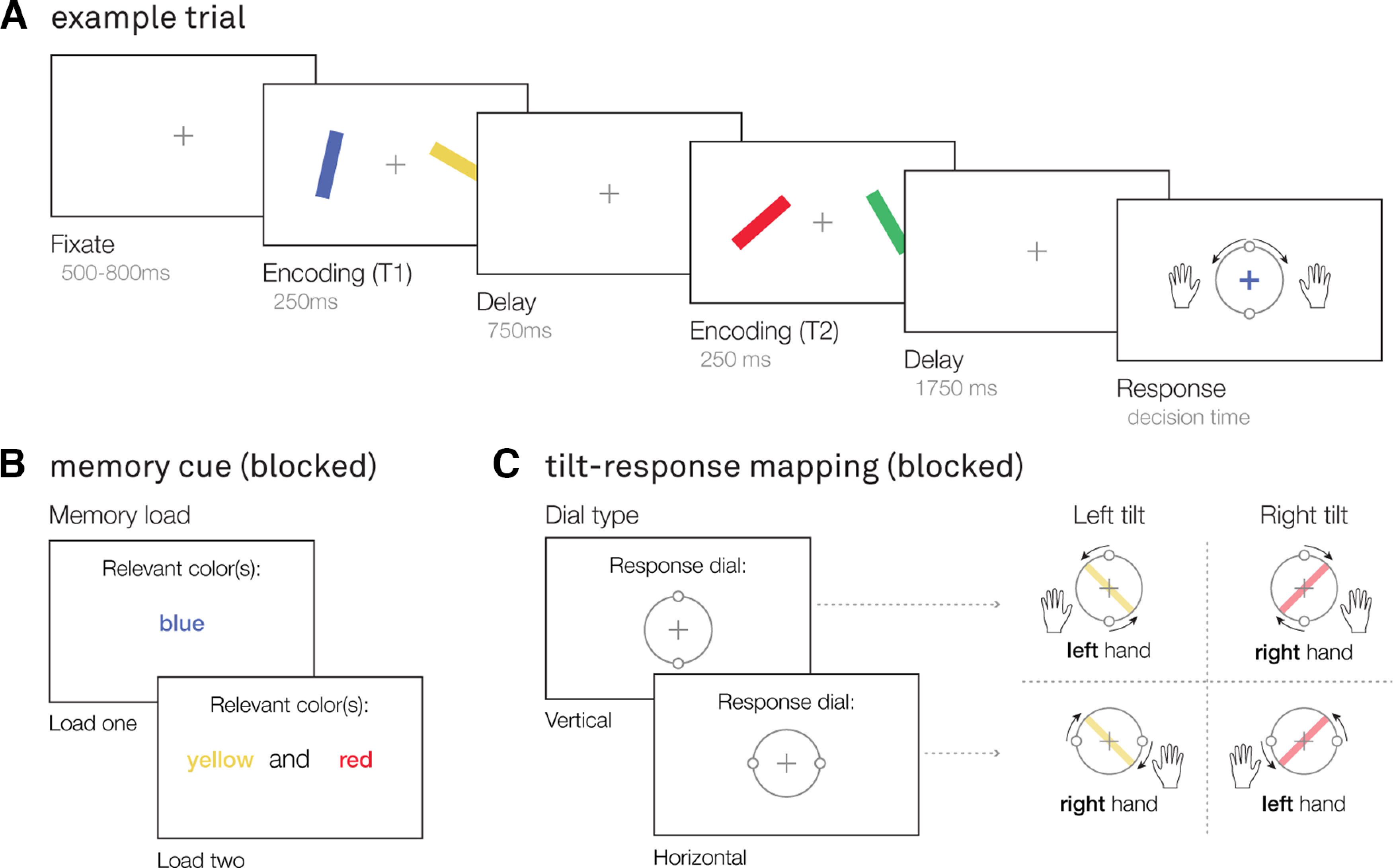
Visual-motor working memory task. ***A***, Example trial. After a brief fixation, participants sequentially viewed two encoding displays, each containing two colored oriented bars, separated by a delay. After a second delay, participants were probed to reproduce the orientation of one of the colored bars using a response dial. The handles of the response dial could be turned clockwise by pressing and holding the “M” key with their right hand, and counterclockwise by pressing and holding the “Z” key with their left hand. ***B***, Memory load manipulation. Preceding a block of trials, participants were instructed to selectively memorize the orientation of one, or two colored bars. In load one, this bar could occur in either of the two encoding displays (early, or late). In load two, each of the two cued bars were presented in a different encoding display (one early, the other late). ***C***, Manipulation of the tilt-response mapping. In addition to memory load, the starting position of the response dial was manipulated between blocks: it could be either vertical, or horizontal. Given that participants could only turn the dial handles a maximum of 90° in either direction, the mapping between tilt and response varied throughout the experiment. Given a vertical response dial, a leftward tilted bar could only be reproduced using the left hand, and a rightward tilted bar using the right hand. Alternatively, given a horizontal response dial, a leftward tilted bar could only be reproduced using the right hand, and a rightward tilted bar using the left hand.

Participants performed a visual-motor working memory task (Fig. 1), in which they were asked to memorize the orientation of colored bars, and reproduce the orientation of one of these bars after a delay using a response dial. We build on a previously developed and used task (van Ede et al., 2019; Boettcher et al., 2021) with three key manipulations.

First, the orientation (leftward or rightward tilt) of the bar was linked to the required response hand, and the mapping between tilt and response hand was counterbalanced by varying the starting point of response dial between blocks (Fig. 1*C*). This uniquely enabled us to disambiguate prospection of the later required response from the visual tilt, since the same tilt was associated with different response hands in different blocks. Second, participants were instructed via a blocked precue to selectively encode one (load one), or two (load two) colored oriented bars (Fig. 1*B*). Crucially, in load one, the memorized orientation was certain to become relevant for behavior during the memory delay, whereas in load two, either of the two bars could potentially become relevant for behavior. This allowed us to test whether prospective responses with less certainty of relevance also evoked action encoding. Third, the cued oriented bars were sequentially presented to the participant in two different encoding displays (Fig. 1*A*). This allowed us to isolate and track action planning for each potential action in load two, and consider the role of this action planning on eventual behavior. We anticipated that planning for the potential action would be particularly clear in response to the first display, given that no prior action-plan could have been formed yet.

The experiment was programmed in PsychoPy (Peirce et al., 2019). All stimuli were presented on a led monitor (ASUS ROG STRIX XG248; 23.8 inch, 1920 × 1080 pixels, 240 Hz) situated 60 cm away from the participant. Participants were instructed to keep central fixation, and their fixation was monitored by the experimenter using an eye-tracker (EyeLink 1000 plus). Each trial contained two separate visual encoding displays (Fig. 1*A*), with two colored oriented bars presented on either side of a fixation cross, at 4° visual angle distance from fixation. One bar was always tilted to the left, the other to the right, and could be presented either to the left or right of the fixation cross. The location of the leftward and rightward oriented bar remained fixed between the two encoding displays during a trial. The magnitude of the orientation of the bars was randomly determined, and varied between 5° and 85° to avoid fully vertical, or fully horizontal orientations. Each bar had a width of 0.4° and height of 4° visual angle, and could have one out of four possible colors: yellow (HEX-value: #C2A025), blue (#3843C2), green (#2FC259), or red (#CF3C3C).

After the second working-memory delay, the fixation cross changed to the color of one the oriented bars, probing participants to reproduce the orientation of the color-matching bar using a response dial. The response dial, presented around fixation, consisted of a large circle with the same diameter as the bar-length with two smaller circles (“handles”) placed on opposite points on the circle, representing an orientation. The position of the response dial handles could be adjusted with a counterclockwise or clockwise turn (max 90° in each direction), by pressing and holding the “Z” or “M” key, respectively. Participants could only press one key per response, and the dial would move clockwise (Z) or counterclockwise (M) for as long as the button was pressed. Participants finalized their orientation report by releasing the chosen key when hitting the desired orientation. Immediately after report finalization, participants received feedback on their precision. This feedback consisted of a number between 0% and 100%, representing the percentage of overlap between the reported and target orientation. Participants were further instructed to respond as fast and as precisely as possible.

We linked visual tilt to the required response hand as follows: a leftward or rightward tilted bar could only be accurately reported by pressing and holding a key with either the left or right hand. Extending our prior studies that leveraged this manipulation (van Ede et al., 2019; Boettcher et al., 2021), we here used two different tilt-response mappings (Fig. 1*C*) that varied across blocks. When the start position of the response dial was vertical (0°), a leftward tilt (relative to the vertical starting point) could only be accurately reported with the left response hand (an “Z” key), and a rightward tilt with the right response hand (an “M” key). When the start position of the response dial was horizontal (90°), a leftward tilt could only be accurately reported with the right response hand, and a rightward tilt with the left response hand (see examples in Fig. 1*C*).

Preceding each block of trials, participants were presented with a precue informing them of the color(s) of the relevant bar(s) that block (Fig. 1*B*). In load-one blocks, participants were instructed to memorize the orientation of a single bar (e.g., only the blue bars' orientation), which was certain to be probed for reproduction report later. In load-two blocks, participants were instructed to memorize the orientations of two bars (e.g., both the yellow and red bars' orientation), which could each potentially be probed for reproduction report (randomly selected, and known to the participant after the memory delay). In both load conditions, target (i.e., the probed item) moment (first, or second encoding display), target location (left, or right side of the screen), and target tilt (left, or right) were counterbalanced within a block. In load two, the nontarget (i.e., the nonprobed item) was always present at a different moment, at a different location, and of a different tilt type.

Preceding the main experiment, participants practiced the task for 5–10 min, or until their performance was good enough (∼75% precision of their orientation-reproduction report). They then completed two consecutive sessions of ∼45 min, with a self-paced break in between. Each session consisted of 12 blocks of 32 trials (384 trials per session). Response dial and memory load type were blocked and counterbalanced, and occurred in a random sequence. The response dial type always remained the same for a minimum of 64 trials. Each dial type block was preceded by four dial-practice trials in which participants practiced turning the response dial clockwise and counterclockwise to match a visible orientation (no working memory component). This was done to avoid confusion about the response dial type during the main trials. These trials were not included in the presented analysis.

### Behavioral analyses

Behavioral data were analyzed using R (R Core Team, 2022). Absolute orientation-reproduction error (in degrees) was defined as the absolute difference in orientation between the target and report. Decision time (in seconds) was defined as the time between probe onset and response initiation. Trials with decision times lower than 100 ms or larger than 5 s were excluded from further analyses. In addition, for each participant, trials were excluded with decision times larger than the mean plus 2.5 times the standard deviation (this was the case for less than ∼3% of the trials). Two one-way repeated measures ANOVAs were performed to evaluate the effect of memory load (one/two), target moment (early/late), and response dial (horizontal/vertical) on the absolute error and decision times. All effects were visualized using the ggplot2 package (Wickham, 2016).

### EEG acquisition and analyses

#### Acquisition

EEG was measured using the BioSemi ActiveTwo System (biosemi.com) with a standard 10–10 System 64 electrode setup. Two electrodes were placed on the left and right mastoid, and used for offline re-referencing of the data. Additionally, EOG was measured using one electrode next to, and another above the left eye.

#### Preprocessing

All EEG analyses were performed in MATLAB (2022a; The MathWorks, 2022) using the FieldTrip toolbox (Oostenveld et al., 2011; https://fieldtriptoolbox.org). Data were epoched from −1000 to 4000 ms, relative to the onset of the first encoding display, and re-referenced to an average of the left and right mastoids. Noisy channels (if present) were interpolated by taking the average of two adjacent electrodes. Next, the data were down-sampled to 200 Hz. Independent component analysis (ICA) was used to correct for blink artifacts. The appropriate ICA components used for artifact rejection were identified by correlating the time courses of the ICA components with those of the measured horizontal and vertical EOG. After blink correction, the FieldTrip function *ft_rejectvisual* was used to visually assess which trials had exceptionally high variance, which were marked for rejection. Trials that had been marked as too fast or too slow (as described above, Behavioral analyses) were also rejected from further analyses. A surface Laplacian transform was applied to increase the spatial resolution of the central motor β signal of interest (as also done by van Ede et al., 2019; Boettcher et al., 2021; Nasrawi and van Ede, 2022).

#### Electrodes, frequency-band, and time-window selection

For all analyses, channel and frequency-band selections were set a priori, and in line with previous research (Salmelin and Hari, 1994; Mcfarland et al., 2000; Neuper et al., 2006; Van Wijk et al., 2009; Boettcher et al., 2021). To investigate motor planning signals contralateral versus ipsilateral to the required response hand, we focused on activity in EEG electrodes C3 and C4. Additionally, we extracted activity in the β band frequency (13–30 Hz) for all our time course visualization.

#### Time-frequency analysis

Time-frequency responses were obtained using a short-time Fourier transform of Hanning-tapered data. A 300-ms sliding time window was used to estimate spectral power between 3 and 40 Hz (in steps of 1 Hz), progressing in steps of 50 ms. Next, activity in motor electrodes (C3/C4) was contrasted between trials in which the required response hand was contralateral versus ipsilateral to these electrodes. This was expressed as a normalized difference: [(contra-ipsi)/(contra+ipsi)] × 100. These contrasts were then averaged across the left and right motor electrodes. To extract time courses of lateralized motor activity, we averaged this contralateral versus ipsilateral response across the 13- to 30-Hz β band. Topographies of the lateralized motor activity were obtained by contrasting trials in which the memory content was associated with a left versus right hand response, expressed as a normalized difference between left and right trials, for each electrode.

Similar to our analysis of lateralized signatures of action planning in canonical motor electrodes (as a function of required response hand), we also analyzed lateralized signatures of visual selection (relative to encoded item location) in canonical visual electrodes PO7/PO8 and zoomed in on the classical (a-priori defined) 8–12 Hz α frequency band.

#### Relation between EEG action-encoding and subsequent behavior

We asked whether the degree of potential-action encoding during the memory delay varied depending on subsequent behavior. To this end, we focused our analysis on both decision times (the onset of memory-guided behavior) and precision (the absolute difference in orientation between the target and response). We hypothesized action planning to pertain particularly to participants' readiness to act on their memory contents. Accordingly, we reasoned action planning was most likely to correlate with subsequent decision times. In addition, with a maximum memory load of two items, report precision was relatively high for this task, and may have been close to ceiling. For this analysis, we focused on load-two trials where we could address whether there may be a trade-off whereby stronger action encoding for one item may benefit performance for that item at the expense of another. To test this, we defined an action-encoding window of interest in terms of frequency and time bounds based on the action-encoding cluster previously observed in load two. Importantly, as this cluster was obtained from all load-two trials, the selection of this window of interest was made regardless of subsequent behavior and target moment. For these extracted data of interest, we then compared trials with fast versus slow decision times after the memory delay (based on a median split), to see whether the pattern of action encoding after visual encoding could predict the speed of onset of subsequent working-memory guided behavior (as in Boettcher et al., 2021). Similarly, for completeness, we compared trials with precise versus imprecise orientation-reproduction reports (based on a median split), to see whether the pattern of action encoding after visual encoding could predict task precision. Here, we did this separately for trials where the probed item occurred in the first or second display. Two one-way repeated measures ANOVA were used to evaluate how the action-encoding differed depending on behavior (fast/slow or precise/imprecise) and target moment (early/late). These effects were visualized using the ggplot2 package (Wickham, 2016).

#### Statistical evaluation

Cluster-based permutations (Maris and Oostenveld, 2007) were performed for the statistical evaluation of the above-described EEG contrasts. This nonparametric approach (or Monte Carlo method) offers a solution for the multiple-comparisons problem in the statistical evaluation of EEG data, which, in our case, included a sizeable number of time–frequency comparisons. It does so by reducing the data to a single metric (e.g., the largest cluster of neighboring data points that exceed a certain threshold) and evaluating this (in the full data space under consideration) against a single randomly permuted empirical null distribution. Cluster-based permutations were performed on the time–frequency responses (considering clusters in time and frequency) and time courses (considering clusters in time) using 10,000 permutations, and an α level of 0.025.

### Code and data availability

The experiment code and analysis code are both publicly available on GitHub. All raw anonymized behavioral and EEG data are available in a public repository on Zenodo.

## Results

### Task performance depends on memory load and is comparable across tilt-response mappings

Before turning to our three main questions of interest (see Introduction), we considered the effects of memory load (one/two), response dial (vertical/horizontal), and target moment (early/late) on task performance. We considered both decision times and errors. Decision time was defined as the time (in seconds) between probe onset and response initiation. Error (in degrees) was defined as the absolute difference between the orientation of the probed memory item and the reported orientation.

**Figure 2.**
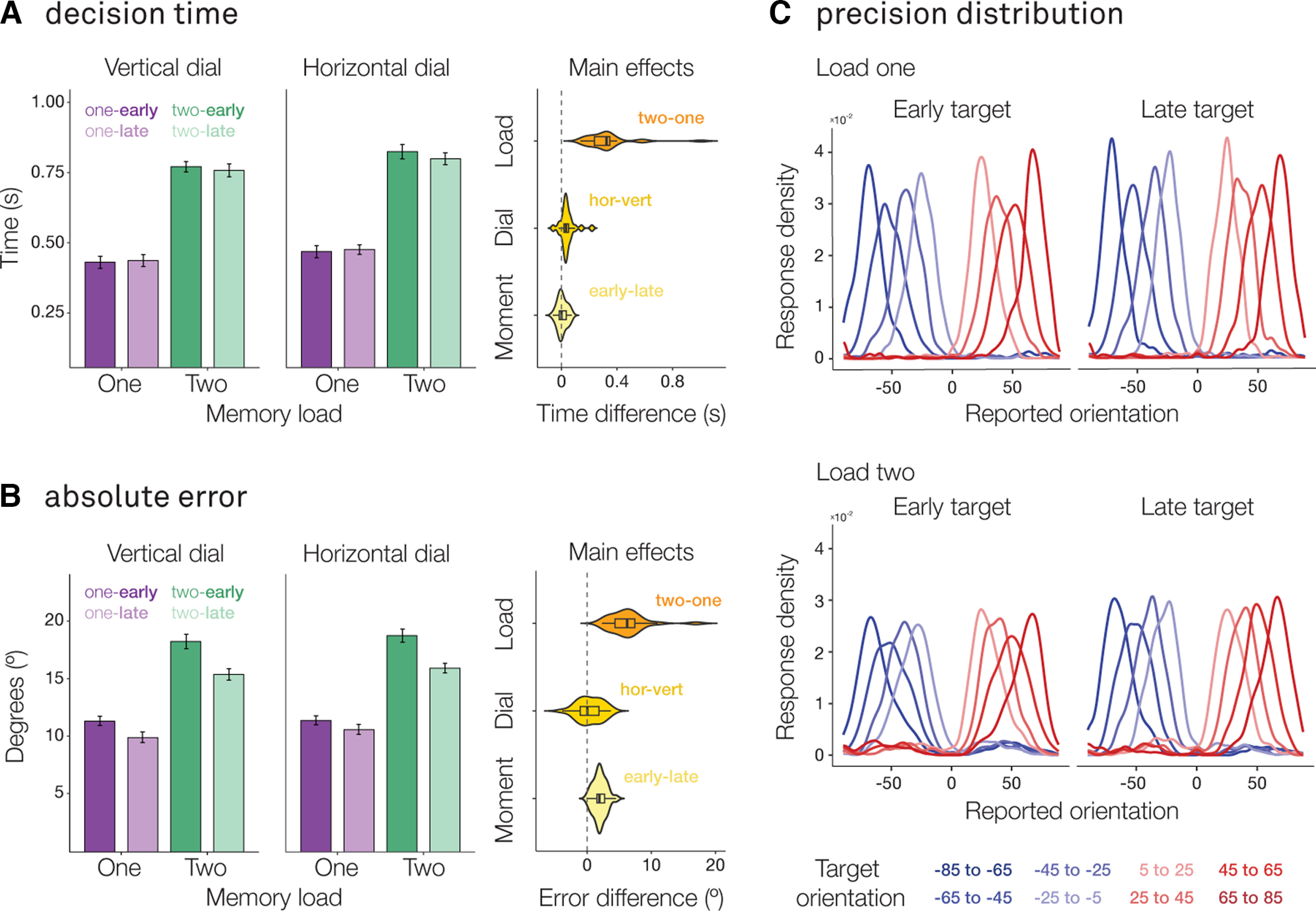
Task performance is comparable across tilt-response mappings. ***A***, ***B***, Bar graphs show the average decision time (in seconds) and absolute error (in degrees) of the orientation-reproduction reports as a function of memory load (one/two), response-dial type (vertical/horizontal), and target moment (early/late). Error bars represent within-participant SE. Violin plots show the main effects of load (two-one), dial (horizontal-vertical), and target moment (early-late). ***C***, Density plot showing the distribution of the reported orientation as a function of the target orientation (in 20° bins), for load one and load two separately, and for early and late targets.

Participants were significantly slower (Fig. 2*A*; *F*_(1,192)_ = 98.92, *p* < 0.001, η^2^ = 0.34) and less precise in their orientation-reproduction report in load two, compared with load one (Fig. 2*B*; *F*_(1,192)_ = 123.50, *p* < 0.001, η^2^ = 0.37). Additionally, we observed that participants were more precise at reproducing the orientation when the probed target item was in the second encoding display (Fig. 2*B*; *F*_(1,192)_ = 12.29, *p* < 0.001, η^2^ = 0.037). However, we did not observe such an effect for decision times (Fig. 2*A*; *F*_(1,192)_ = 0.034, *p* = 0.854, η^2^ = 0.0001). Importantly, we observed no effect of response-dial type on either decision time (*F*_(1,192)_ = 1.638, *p* = 0.202, η^2^ = 0.006) or absolute error (*F*_(1,192)_ = 0.621, *p* = 0.431, η^2^ = 0.002), suggesting task difficulty was comparable across tilt-response mappings. We also observed no significant interactions between any of the variables on either measure of task performance (all *p* > 0.05).

Finally, reported-orientation distributions for different target orientations (Fig. 2*C*) revealed how participants guided their orientation-reproduction reports by the precise visual orientation of the probed memory item, rather than merely remembering whether to press the left or the right button at the end of the memory delay. This is apparent both in load-one (upper panel) and in load-two (lower panel), as well as for early (left plot) and late (right plot), targets. Additionally, these also confirm the reduced overall precision in load two (quantified above).

### Action encoding flexibly codes for the anticipated task

We now turn to our first main question: do we find action encoding even when the tilt-response mapping is varied across blocks (i.e., the same visual tilt is associated with different response hands in different blocks, as shown in Fig. 1*C*)?

An established neural marker of manual-action planning in the EEG signal is the attenuation of β band activity in electrodes contralateral versus ipsilateral to the required response hand (Salmelin and Hari, 1994; Mcfarland et al., 2000; Neuper et al., 2006; Van Wijk et al., 2009), including in the context of prospective visual-working-memory-guided behavior (Schneider et al., 2017; Boettcher et al., 2021; Ester and Weese, 2023; Nasrawi and van Ede, 2022; Rösner et al., 2022). Here, we tracked this action-planning signal during the memory delay for each memory load condition (one/two). In load one, we did so separately for early and late targets (occurring either in the first, or second encoding display). In load two, the two potential targets were presented across the two encoding displays, and always required a different response hand. Accordingly, we could collapse over the early and late target trials, and defined contralateral versus ipsilateral relative to the required response hand associated with the target-tilt in the first encoding display.

**Figure 3.**
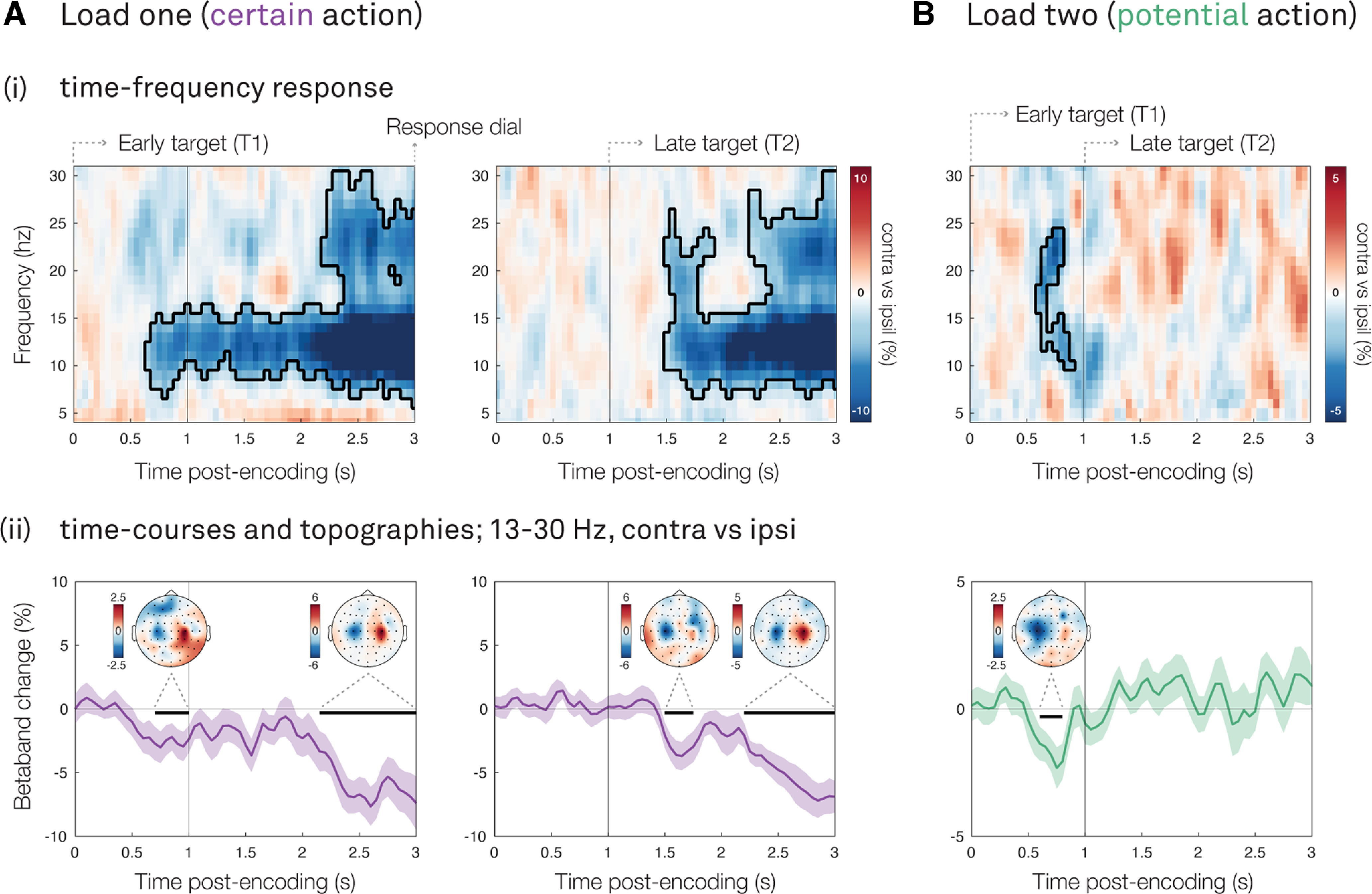
Action encoding flexibly codes for the anticipated task, and even when this is a potential task. For (***A***) load-one (certain action) and (***B***) load-two (potential action) trials: ***i***, Lateralized motor activity relative to the response hand that is required to reproduce the visual item, calculated in canonical motor electrodes (C3/C4). The black outlines indicate significant clusters yielded from cluster-based permutation analysis. The vertical black lines indicate the onset of the encoding displays. ***ii***, Time courses of lateralized motor activity at 13–30 Hz (as in Boettcher et al., 2021). The black horizontal lines indicate significant clusters yielded from cluster-based permutation analysis. The vertical black lines indicate the onset of the encoding displays. Topographies show associated 13- to 30-Hz motor lateralization (left vs right required response hand), for each significant time course-cluster. Shadings indicate SE across participants.

In line with previous studies (Schneider et al., 2017; Boettcher et al., 2021; Nasrawi and van Ede, 2022), we show a clear attenuation of β band power in motor electrodes contralateral versus ipsilateral to the required response hand when the planned action is certain to become relevant (i.e., load one; Fig. 3*Ai*; early target T1, cluster *p* < 0.0001; late target T2, cluster *p* = 0.0001). As seen in the β band time courses (Fig. 3*Aii*), these effects arise early after the onset of the relevant visual encoding display (early target T1, cluster *p* = 0.04; late target T2, cluster *p* = 0.04). This is in line with an early “encoding” of the prospective action (as in Boettcher et al., 2021) that is certain to become relevant after the memory delay. Following this initial early postencoding lateralization, we observed another clear attenuation of β band power preceding the memory probe, in anticipation of response execution (early target T1, cluster *p* < 0.0001; early target T2, target cluster *p* = 0.0001). Topographies, showing the difference between trials that required a left-hand or right-hand response, confirmed that these motor signatures were particularly prominent in the corresponding left and right motor electrodes (C3/C4). This was the case both early after visual encoding, and before the probe-onset (Fig. 3*Aii*).

The data from the load-one condition alone already provide an important advance over previous studies that linked visual features of memory content to manual actions (Schneider et al., 2017; van Ede et al., 2019; Boettcher et al., 2021; Ester and Weese, 2023; Henderson et al., 2022; Nasrawi and van Ede, 2022; Rösner et al., 2022). By varying the tilt-response mapping we could isolate action planning signatures from signatures associated with specific visual tilts because in half the blocks the same visual tilt would be associated with the opposite response hand (for details, see Materials and Methods). Accordingly, our observed action encoding (Fig. 3*A*) cannot be because of a fixed automatic mapping between visual tilt and a manual action. Instead, it must be attributed to flexible prospection of the upcoming task demand.

### Action encoding occurs for both certain and potential prospective actions

We now turn to our second main question: are, in addition to certain future actions, potential future actions also prospected when sequentially encoding multiple visual items?

To address this question, we turned to the data from our load-two condition. Strikingly, we observed a similar lateralization of β band power after visual encoding when the planned action could potentially become relevant, as was the case in load two (Fig. 3*Bi*; cluster *p* = 0.01). Here, the chance that the encoded item would be probed for report was only 50%. Similar to what we observed in load one, this signature was apparent early after the onset of the first visual encoding display (time course cluster *p* < 0.02), and was again associated with a modulation in canonical motor electrodes (Fig. 3*Bii*).

Thus, in addition to certain actions, potential actions are encoded immediately after sensory encoding (referred to as “output planning at the input stage” in Boettcher et al., 2021). This suggests that the brain prospects a potential future manual action that is associated with a visual stimulus, even when this action might become relevant after the memory delay.

### Action encoding facilitates performance speed at the expense of subsequent memory items

Next, we considered how potential-action encoding relates to subsequent memory-guided behavior at the end of the memory delay: does stronger encoding of a potential future action benefit subsequent behavior? And if so, does it come at the expense of other memory items?

**Figure 4.**
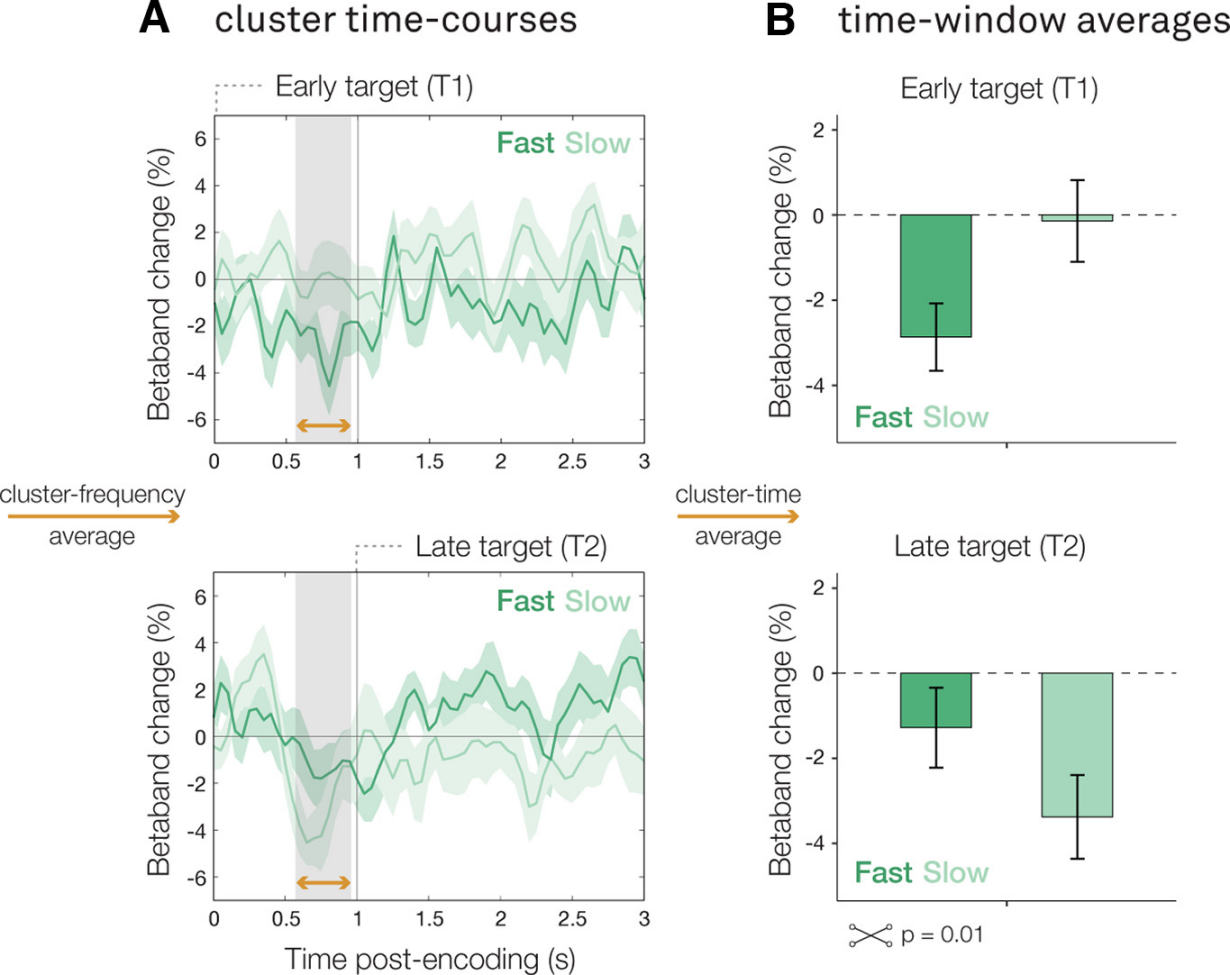
Stronger potential action encoding facilitates performance speed at the expense of subsequent memory items. ***A***, Time course of the lateralized motor activity cluster observed in load two, calculated in canonical motor electrodes (C3/C4). ***B***, Bar graphs of the lateralized motor activity cluster observed in load two. Top panels show the response in trials where the target occurred in the first encoding display (T1, early). Lower panels show the response in trials where the target occurred in the second encoding display (T2, late). Dark green time courses represent trials that ultimately had fast decision times; light green time courses represent trials that ultimately had slow decision times; trials were marked as fast/slow using a median split. Error bars represent within-participant SE.

To test this, we zoomed in on the potential-action encoding effect in load two (Fig. 3*Bi*), where there were two items that competed in working memory. We averaged over the frequency band (Fig. 4*A*) and time window (Fig. 4*B*) of the observed potential-action encoding cluster. We then looked at β lateralization in this time-frequency window (as a marker for the degree of action encoding) and compared trials with fast versus slow decision times, separated using a median split, as well as trials with precise versus imprecise orientation-reproduction reports. Crucially, we did this separately for trials where the probed item was an early (T1) versus a late (T2) target.

This revealed a significant interaction between the moment of the probed item (early/late target) and the speed of memory-guided behavior (fast/slow) regarding the degree of potential-action encoding after the first encoding display (*F*_(1,96)_ = 6.84, *p* = 0.01). The pattern of this interaction is consistent with a performance trade-off: when participants were probed to reproduce the orientation of the item in the first display (Fig. 4, top panels), they were faster if they had more strongly encoded the action associated with this item (i.e., more β lateralization in our action-encoding time-window of interest). However, if they were subsequently probed to reproduce the orientation of the item in second encoding display (Fig. 4, bottom panels), participants responded slower after having more strongly encoded the potential action associated with the visual item in the first encoding display.

**Figure 5.**
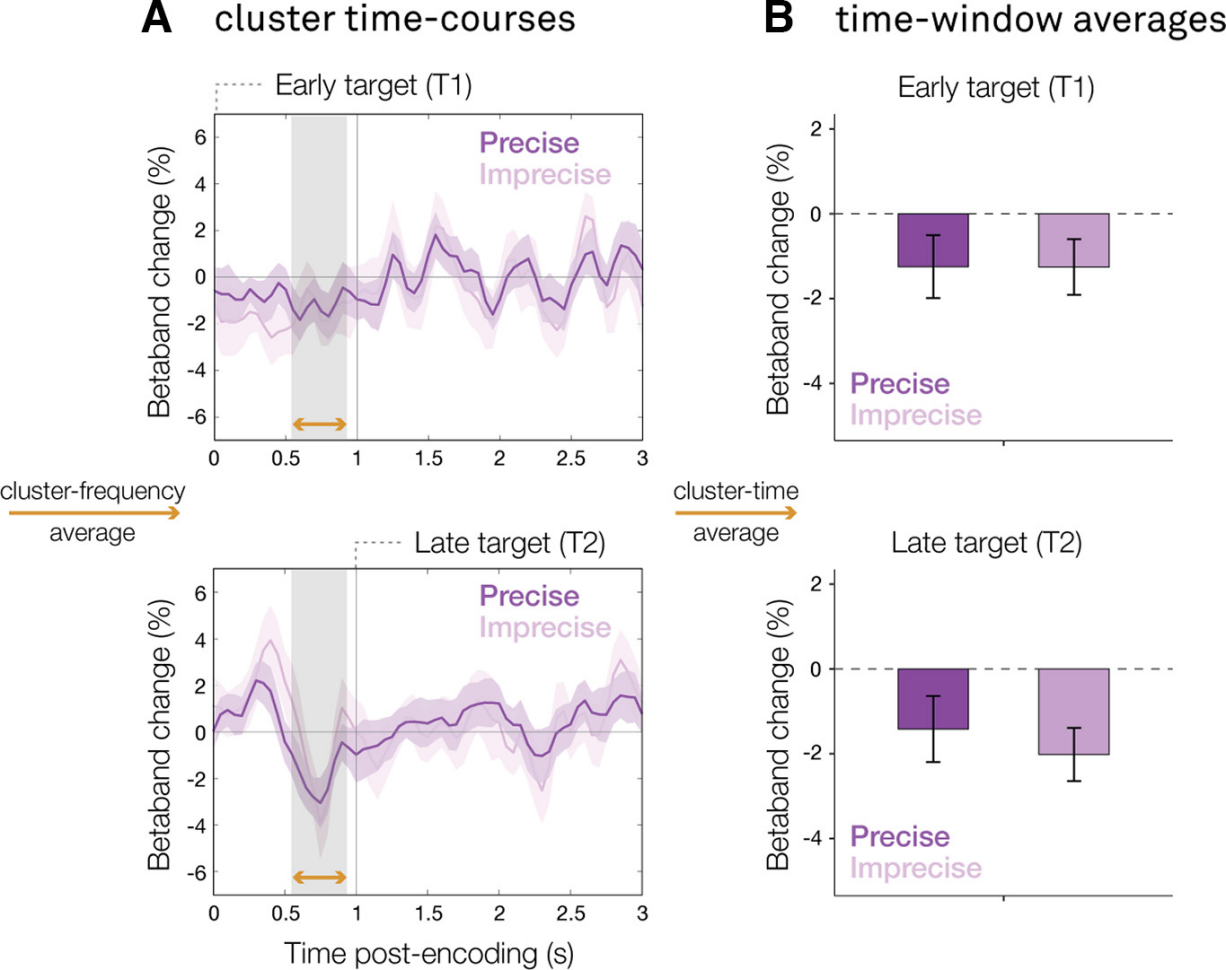
No evidence for a relation between action encoding and task precision. ***A***, Time course of the lateralized motor activity cluster observed in load two, calculated in canonical motor electrodes (C3/C4). ***B***, Bar graphs of the lateralized motor activity cluster observed in load two. Top panels show the response in trials where the target occurred in the first encoding display (T1, early). Lower panels show the response in trials where the target occurred in the second encoding display (T2, late). Dark purple time courses represent trials that ultimately had high task precision; light purple time courses represent trials that ultimately had low task precision; trials were marked as precise/imprecise using a median split. Error bars represent within-participant SE.

Although we only hypothesized to find a relation between decision times and the degree of action encoding (see Materials and Methods), for completeness we performed the same median split analysis as we used for decision times for recall precision (precise/imprecise). This did not yield any significant effects (Fig. 5).

In other words, stronger encoding of a potential future action facilitates the speed of later memory-guided behavior for the associated memory content, but this comes at the expense of responding to other potentially relevant and subsequently encoded working-memory content. Additionally, we find no evidence for a similar relation between action encoding and task precision.

### Similar visual selection of the first and second potential target item

Because of the way we designed our experiment (as justified in Materials and Methods), the first relevant item in load-two trials (T1) provided predictive information about the second item (T2). Specifically, it could serve as a cue for its location and required response hand (but crucially not the exact tilt). This could have incentivized participants to attend to the first item more strongly, or even to infer the second item without attentively encoding it.

The observed behavioral response distributions argue against this possibility, as we found reports that matched the detailed tilt of T2 (Fig. 2*C*), while the exact tilt of T2 was never known in advance. Here, we provide additional neural evidence that participants actively selected the second target in load-two trials. To do so, we tracked lateralization of α band activity in visual electrodes (PO7/PO8) relative to the item locations, a canonical neural measure of visual-spatial attention (Sauseng et al., 2005; Thut et al., 2006; Worden et al., 2000).

**Figure 6.**
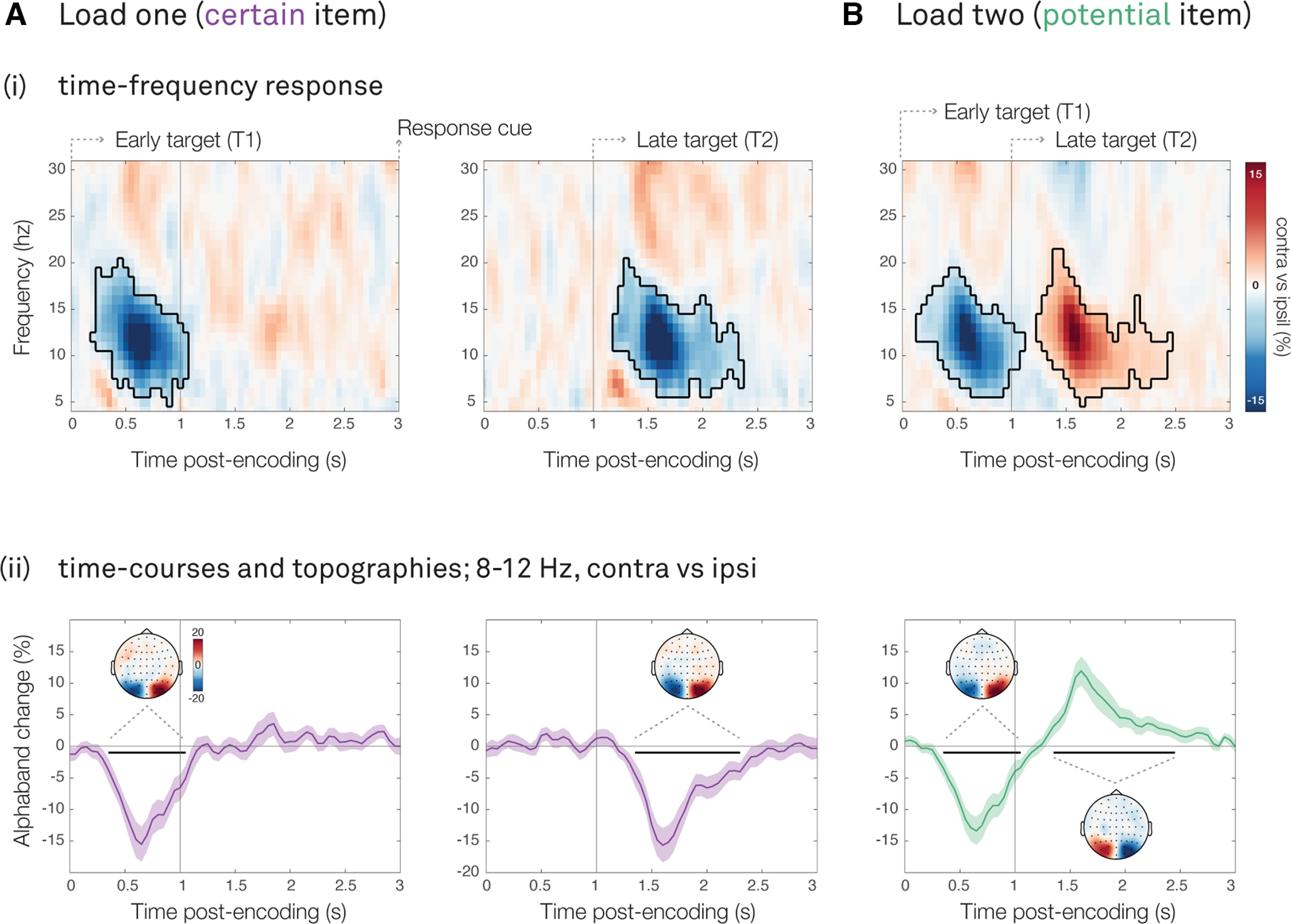
Visual encoding of both certain and potential items. For (***A***) load-one (certain item) and (***B***) load-two (potential item) trials: ***i***, Lateralized visual activity relative to the item location, calculated in canonical visual electrodes (PO7/PO8). The black outlines indicate significant clusters yielded from cluster-based permutation analysis. The vertical black lines indicate the onset of the encoding displays. ***ii***, Time courses of lateralized visual activity at 8–12 Hz (as in Boettcher et al., 2021). The black horizontal lines indicate significant clusters yielded from cluster-based permutation analysis. The vertical black lines indicate the onset of the encoding displays. Topographies show associated 8- to 12-Hz visual lateralization (left vs right item location), for each significant time course-cluster. Shadings indicate SE across participants. Note: for load two-T2, the α-lateralization reverses given that the contrast is defined relative to the location of T1, and the location of T2 was always opposite (i.e., left, if T1 was located on the right).

For reference we again first considered load-one trials. We observed a clear attenuation of α band power in visual electrodes contralateral versus ipsilateral to the location of the target memory item (Fig. 6*Ai*), both for early (left panel, cluster *p* < 0.0001) and late targets (right panel, cluster *p* < 0.0001). As seen in the averaged 8- to 12-Hz α band time courses (Fig. 5*Aii*), these effects arise early after the onset of the visual encoding display (early target T1, cluster *p* < 0.0001; late target T2, cluster *p* < 0.0001). Topographies, showing the difference between trials with the relevant item on the left versus right side of the display, confirmed that these visual signatures were most prominent in the left and right posterior EEG electrodes (PO7/PO8).

In load-two trials (Fig. 6*Bi*), we also observe clear attenuation of α band power in visual electrodes contralateral versus ipsilateral to item location. Critically, this occurred following both the early target (T1, cluster *p* < 0.0001) and the late target (T2, cluster *p* < 0.0001), with similar patterns following either encoding display. Note that because the contrast was defined by the location of the early target (T1), the lateralization reverses for the oppositely located late target (T2). As shown in the averaged α band time courses (Fig. 6*Bii*), these effects arise early after the onset of each encoding display (early target T1, cluster *p* < 0.0001; late target T2, cluster *p* < 0.0001), and were again prominent in the left and right posterior electrodes (PO7/PO8).

These data provide neural evidence that participants actively selected both the early and late target item, despite the location of the first item providing predictive information about the location of the second item. This is consistent with the instruction to report the exact tilt of either T1 or T2, which required participants to attend to both items.

## Discussion

It is increasingly studied how visual working memory helps us to prepare for the future, in addition to how it helps us to hold onto the past (Rainer et al., 1999; Myers et al., 2017; van Ede and Nobre, 2023). In line with this perspective, many recent studies have revealed close links between visual working memory and action (for review, see Heuer et al., 2020; Olivers and Roelfsema, 2020; van Ede, 2020). For instance, it has been shown that action planning takes place alongside retention of visual representations in working memory (Schneider et al., 2017; van Ede et al., 2019; Boettcher et al., 2021; Ester and Weese, 2023; Henderson et al., 2022; Kikumoto et al., 2022; Nasrawi and van Ede, 2022; Rösner et al., 2022; Trentin et al., 2023). Specifically, Boettcher et al. (2021) demonstrated that such output planning may already commence at the input stage, a pattern that was referred to as “action encoding.” The current study advances this growing body of research in at least three ways.

First, we show that action planning – as reflected by the pattern of action encoding during the memory delay, truly reflects the early prospection of a future required task, as it flexibly adapts to different visual-motor mappings. Similar to previous studies (Schneider et al., 2017; van Ede et al., 2019; Boettcher et al., 2021; Henderson et al., 2022; Rösner et al., 2022), we linked visual tilt (leftward or rightward) to specific manual responses. Critically, however, in the current study we additionally varied the mapping between tilt and response hand, such that any specific tilt required reproduction with a left hand in some blocks, and with the right hand in other blocks. Despite this manipulation, we still observed clear action encoding when considering EEG motor signals contralateral versus ipsilateral relative to the required response hand (and thus over and above the visual tilt). This shows that action encoding cannot reflect an automatic response to a specific visual orientation, but instead reflects a genuine prospection of the future task that is required to reproduce that orientation.

Second, we show that early action encoding occurs not only when this action is certain to become relevant (as in our load-one condition; and as previously shown by Boettcher et al., 2021), but also when this action is potentially relevant for future behavior. In everyday life, we are often required to encode and retain multiple visual items in working memory that each afford different actions, of which only some may become relevant for behavior depending on how the situation unfolds. Indeed, a key function of working memory may be to enable us to be ready for multiple potential courses of action (Cisek and Kalaska, 2005, 2010; van Ede et al., 2019; Nasrawi and van Ede, 2022; van Ede and Nobre, 2023). In the current study, we show that the observation of “output planning at the input stage” (Boettcher et al., 2021) extends to the planning of potential outputs: action encoding occurs even when participants know they will have to encode another item into working memory that is equally likely to become relevant for behavior after the memory delay. Thus, even when an action might only potentially become relevant in the future, it is still prospected immediately after visual encoding.

Third, by considering action encoding in a situation with multiple items in memory, we were in a unique position to reveal that stronger potential action encoding for one item may come at the expense of another. Specifically, we observed a performance speed trade-off, where stronger action encoding for one item is beneficial to decision times when this item is subsequently probed, but hampers decision times when the other item is probed. This reveals that there is variation in the degree to which a potential action is encoded, that is, potential actions may be more or less strongly prioritized, which is reflected in performance later on. This is consistent with recent research showing that actions that were made or planned during visual working memory can affect performance of action-matching items, at the expense of other items (Hanning et al., 2016; Heuer and Schubö, 2017, 2018; Ohl and Rolfs, 2017, 2018, 2020; Hanning and Deubel, 2018; Trentin et al., 2023).

In our load-one condition, we observed action encoding both when the relevant item occurred in the first and second display (Fig. 3). Interestingly, however, in our load-two condition, we only observed action encoding following the first display, with little evidence for a similar effect following the second display. Different factors may account for this. In our design, the relevant items in the first and second display were always linked to different response hands. In our analysis, we focused on lateralization of β band power relative to the required response hand in the first display. After the first encoding display, only one potential action could be planned (reflected in the observed “potential action encoding” effect). In contrast, after the second display, participants were required to add a second potential action, while maintaining the action associated with the first display. Accordingly, the lack of clear potential action encoding following the second display may be because of both potential actions balancing out relative β band change, as they each require a different response hand. Alternatively, the first potential action may be selectively planned, while the second one is ignored, or the second item may be attended to a lesser extent. However, this alternative explanation would predict a clear behavioral benefit for the item in the first display, but we found no evidence for this, neither for decision times, nor for precision (Fig. 2*A*,*B*, main effects). In addition, neural evidence speaks against the possibility that the second item may be attended to a lesser extent, as we observed clear visual selection of both items in load two, as reflected by the lateralization of posterior α activity (Fig. 5*B*).

In our paradigm, the encoding of multiple visual items into working memory occurred sequentially. This manipulation allowed us to track action encoding for each memory item separately, advancing previous research where multiple visual items were presented at the same time (van Ede et al., 2019; Nasrawi and van Ede, 2022). Yet, together with the advance of this manipulation, we also lost the possibility to infer whether multiple potential actions were planned and encoded in parallel. From dedicated action-planning studies, there is evidence that multiple actions can be planned in parallel, before selecting the relevant action for implementation (Cisek and Kalaska, 2005; Cisek, 2007; Grent-'t-Jong et al., 2013; Gallivan et al., 2015, 2016; but cf. Dekleva et al., 2018). Whether this also holds for the encoding of multiple potential actions (i.e., parallel action encoding) in the context of visual working memory tasks remains an exciting avenue for future research.

In summary, our findings confirm that action planning, and specifically action encoding, alongside visual working memory reflects anticipated task demands, rather than an automatic response to visual tilt; occurs even for actions that will potentially become relevant for behavior; and is associated with faster performance, at the expense of performance to other memory content. Together, these results demonstrate that the potential prospective use of visual working memory content is flexibly planned for early on, with consequences for later performance.
